# Reference values of body composition parameters and visceral adipose tissue (VAT) by DXA in adults aged 18–81 years—results from the LEAD cohort

**DOI:** 10.1038/s41430-020-0596-5

**Published:** 2020-03-02

**Authors:** Alina Ofenheimer, Robab Breyer-Kohansal, Sylvia Hartl, Otto C. Burghuber, Florian Krach, Andrea Schrott, Emiel F. M. Wouters, Frits M. E. Franssen, Marie-Kathrin Breyer

**Affiliations:** 1 NUTRIM, School of Nutrition and Translational Research in Metabolism, Maastricht University Medical Center, Maastricht, the Netherlands; 2 Ludwig Boltzmann Institute for COPD and Respiratory Epidemiology, Vienna, Austria; 3 Medical School, Sigmund Freud University, Vienna, Austria; 4 Department for Respiratory and Critical Care Medicine, Otto Wagner Hospital, Vienna, Austria; 5 Department of Mathematics, ETH Zurich, Zurich, Switzerland; 6 Department of Research and Education, CIRO, Horn, the Netherlands

**Keywords:** Epidemiology, Diagnosis

## Abstract

**Background:**

Increasing attention has been drawn on the assessment of body composition phenotypes, since the distribution of soft tissue influences cardio-metabolic risk. Dual-energy X-ray absorptiometry (DXA) is a validated technique to assess body composition. European reference values from population-based cohorts are rare.

**Aims:**

To provide age- and sex-related reference values of body composition parameters and visceral adipose tissue (VAT) mass, and for lean mass index (LMI) with regard to fat mass index (FMI) quantities and BMI categories.

**Methods:**

GE-Lunar Prodigy DXA scans of 10.894 participants, aged 18–81 years, recruited from 2011 to 2019 by the Austrian LEAD study, a population-based cohort study, have been used to construct reference curves using the LMS method. Parameters assessed are FMI, LMI, appendicular LMI, fat mass ratios android/gynoid and trunk/limbs, and VAT.

**Results:**

All lean mass and fat mass parameters indicating central fat accumulation were higher in men, whereas other fat mass indices were higher in women. LMI differed between each FMI subgroup (low vs. normal, low vs. high, normal vs. high), and BMI category in all ages and LMI increased with FMI and BMI classes. VAT mass was higher in men compared with women and increased across all age groups within both sexes.

**Conclusion:**

The present study provides age- and sex-related reference values for European adults aged 18–81 years for body composition parameters and VAT mass for Lunar Prodigy DXA. In addition, this study reports LMI reference values with regard to fat mass quantities, showing a positive association with increasing FMI percentiles and BMI categories.

## Introduction

The human body is often described as a three-compartment model, consisting of the tissue components fat mass (FM), lean mass (LM), and bone mineral content (BMC) [1], with individually different proportions, resulting in diverse body composition phenotypes. Dual X-ray absorptiometry (DXA) is a validated tool to investigate body composition phenotypes [2–4] as it precisely analyses the amount of BMC and soft tissue (FM and LM) of the whole body and specific anatomical regions [1, 5]. A recently developed application (CoreScan^TM^, GE Healthcare^©^, USA) of the DXA scanner can estimate visceral adipose tissue (VAT) fat, based on measurements of VAT mass and volume in the android region, which is defined by the DXA automatically as a region-of-interest, located above the iliac crests with a height of 20% of the distance between the iliac crest and the skull base [6–9]. In the last years, increasing attention has been drawn on the assessment of body composition phenotypes, since the distribution of soft tissue, in particular FM, negatively influences the cardio-metabolic risk of individuals [10, 11]. It is well known that the relationship between obesity and cardiovascular, as well as metabolic morbidities, depends on the distribution of FM rather than on its total amount [11]. Abdominal adiposity, especially an excessive amount of VAT, is associated with insulin resistance, diabetes mellitus type 2, hypertension, dyslipidaemia, and cardiovascular disease (CVD) [11, 12], and even increased all-cause mortality [13–15]. Fat accumulation in the gluteal, femoral, and peripheral leg regions, however, is associated with a better plasma lipid profile [12], indicating a lower risk for the development of CVD. Regional fat distribution can be determined by anthropometric measures such as waist-, hip-, arm- or thigh circumferences as well as their ratios. However, it has been shown that the assessment of FM by DXA is a better predictor for risk factors of comorbidities than the conventional anthropometric measurements [16].

Fat-free mass is composed of BMC and LM. The latter, in particular when measured at the limbs, is a marker of skeletal muscle mass and therefore important in the assessment of muscle depletion [17]. Subjects having reduced LM or increased FM values can be identified by comparison with appropriate reference populations. Recently, it has been emphasised, that for LM reference values its relationship with FM has to be taken into account [17] since body composition changes and diversities affect both tissue components simultaneously. However, available reference values for LM do not account for FM quantities so far.

To be mentioned, results from different manufacturers are not directly interchangeable, and even between DXA devices from the same manufacturer, differences have been reported [18]. Therefore, the used DXA systems have to be considered when comparing reference values. In addition, the WHO reports significant geographical differences in the prevalence of overweight [19], and it is known that substantial variations of body composition exist between populations [20], reinforcing the need for reference values based on different geographical areas.

Therefore, the present analysis aims to provide in a large European population-based cohort, aged 18–81 years, age- and sex-related reference values of total and regional body composition parameters, VAT mass, as well as reference values for lean mass index (LMI) with regard to FMI quantities and to BMI categories by Lunar Prodigy DXA.

## Materials and methods

### Study design

The LEAD Study (clinical trial number: NCT01727518) is a single-centre, longitudinal, observational, and population-based cohort study. It investigates a random sample of Caucasian participants, recruited from the general population of Austria stratified by age, sex, and residential area (based on the inhabitants’ register). Comparison with the governmental data of Austria showed almost identical distribution of age and gender, supporting the representativeness of the LEAD cohort for the Austrian population [21]. Further details concerning the objectives, methodology, and external validity of the LEAD study can be found elsewhere [21].

### Subjects

For this analysis, all adult participants (aged 18–81 years) with valid whole-body DXA scans, from 2011 to 2019, were included. Exclusion criteria involved being pregnant or currently breastfeeding. At the manufacturer’s request, subjects having a body weight exceeding the limit of 159 kg were further excluded, and for VAT analysis only adults having a BMI within the range of 18.5–40 kg/m^2^ could be included.

### Measurements

#### Anthropometrics/body composition

The BMI was calculated as weight/height^2^ (kg/m^2^). Whole-body scans were conducted with a Lunar Prodigy^TM^ (GE Healthcare^©^, USA) DXA scanner. Body composition parameters were analysed using the software enCORE^TM^ (version 17, 2016), and VAT was measured with the CoreScan^TM^ (GE Healthcare^©^, USA). Each day a quality control was conducted, and calibration of the model was performed, following the instruction protocol provided by the manufacturer. Participants were told to remove all heavy metal objects, such as jewellery and watches. Shoes, jeans, and all clothes containing zippers or press buttons, as well as bras containing a wire, had to be taken off to as well. All participants were examined after completing a fasting period of at least 8 h.

For this analysis, the variables of interest derived from the DXA dataset are FM (kg), LM (kg) of the whole body and of defined anatomical regions (limbs, trunk, android, and gynoid) and visceral adipose tissue (VAT mass, g and VAT volume, cm^3^). In addition to the measurement of VAT, the ratio FM android/gynoid was calculated as an indirect marker of abdominal fat accumulation, due to its broader availability.

Additional parameters were calculated as following:

- FMI: fat mass/height^2^ (kg/m^2^)
- LMI: lean mass/height^2^ (kg/m^2^)
- appendicular LMI: lean mass of four limbs/height^2^ (kg/m^2^)
- appendicular FMI: fat mass of four limbs/height^2^ (kg/m^2^)
- FM trunk/limbs: fat mass trunk/fat mass limbs (kg)
- FM android/gynoid: fat mass android/fat mass gynoid (kg)

The coefficient of variation (CV) was calculated as root mean square standard deviation divided by the mean. The %CV of the DXA scanner, all measurements were taken with, was 1.15% for total FM, 0.71% for total LM, 1.33% for %FM, 0.68% for %LM, 3.80% for FM android, 2.84% for FM gynoid, 2.82% for FM trunk, 2.53% for FM limbs, and 2.12% for appendicular LM.

### Statistics

The dataset was stratified by sex, and for analysis of descriptive statistics, it was further divided into age groups (18 to <30, 30 to <40, 40 to <50, 50 to <60, 60 to <70, and 70 to <82 years). The statistical significance level was set to *p* < 0.01 and Bonferroni–Holm multiple testing correction (MTC) was applied for *p* values, whenever necessary. Each parameter was tested for significant differences between the different age groups. Furthermore, for each parameter and age group, males and females were tested for significant differences. The testing was done with the following procedure. First, all groups were checked for normality (N, using Shapiro–Wilk test) and whether they have equal variance (EV, using Levene test). Depending on the outcomes of these two tests, the test to compare the groups was chosen:

- N = yes, EV = yes: standard ANOVA (analysis of variance) by linear model fitting with post-hoc Scheffe test (test between age groups); *T*-tests with equal variance assumption and MTC (test between sex)
- N = yes, EV = no: ANOVA with Kruskal–Wallis test with post-hoc *T*-tests without equal variance assumption with MTC (test between age groups); *T*-tests without equal variance assumption with MTC (test between sex)
- N = no: ANOVA with Kruskal–Wallis test with post-hoc Mann–Whitney *U*-tests with MTC; Mann–Whitney *U*-tests with MTC (test between sex)

Percentile curves for body composition parameters were created using the LMS method described by Cole and Green [22]. First, this statistical method estimates the optimal power in the Box-Cox transformation to obtain normality of the transformed data (*L*), the median (*M*), and the CV (*S*). They are estimated as smooth parameter curves depending on a specific variable, e.g. age. The degrees of freedom of the L, M, and S curves were chosen by fitting multiple models and selecting the best using the BIC (Bayesian Information Criterion) criterion. Extreme outliers (standardised residuals >10) were identified using a linear model and excluded from the LMS-model fitting process to obtain models with a good fit. However, this exclusion criterion was only applied in one case for the FM trunk/limbs model. The parameter curves (L, M, and S) of the selected LMS-model were used to construct percentile curves (3rd, 10th, 50th, 90th, and 97th) for the original data. By using the variable-specific parameters, *L*, *M*, and *S*, individuals’ body composition *z*-scores of each parameter can be calculated by the following formula: $z\,=\,\frac{{[ {\frac{y}{{M\left( t \right)}}}]^{L\left( t \right)} - 1}}{{L\left( t \right)S(t)}}.$

To check whether the selected model yields reasonable percentiles, we calculated the percentage of data points lying below each percentile curve and found acceptable deviations. Moreover, we checked whether *z*-scores calculated with the L, M, and S values published in this article correspond to a standard Gaussian distribution by the Kolmogorov–Smirnov test (*p* < 0.05, using MTC). This was found to be true for all parameters evaluated. For the LMI models stratified by FMI, we first created the FMI model and split the sample according to the 25th and 75th percentile based on this model. In the next step, we created LMI models for each subgroup (low FMI: 0 to ≤25th percentile, normal FMI: >25th to <75th percentile, high FMI: ≥75th percentile). Subgroups were compared following the same procedure as described above for age group comparisons. In addition, LMI models were created based on participants of different BMI categories. Therefore, participants were stratified based on their BMI according to WHO BMI categories (18.5 to <25 kg/m^2^, 25 to <30 kg/m^2^, and ≥30 kg/m^2^) before fitting a model for the LM parameters LMI and appendicular LMI. For the BMI category indicating underweight (<18 kg/m^2^), the respective sample size was too small to create representative LMI percentile curves. BMI subgroups were again compared following the same procedure as described above for age group comparisons.

All statistical analyses were performed using R (2004–2016 The R Foundation for Statistical Computing http://www.R-project.org). The LMS models were calculated using the R package VGAM (https://cran.r-project.org/web/packages/VGAM/index.html). The R code will be published on github (https://github.com).

## Results

### *Subjects* characteristics

In total, 10.894 participants (5.147 men vs. 5.747 women) aged 18–81 years were included in this analysis, in 10.299 (5.025 men vs. 5.274 women) of whom VAT was analysed. Table 1 shows the characteristics of the study sample.

**Table 1:** Descriptive characteristics of study population.

	Age (yrs)						
	18 to <82	18 to <30	30 to <40	40 to <50	50 to <60	60 to <70	70 to <82
Men							
*n*	5147	1233	885	830	839	784	576
Height (cm)	177.6 ± 7.1	178.6 ± 7.1	178.8 ± 6.9	179.2 ± 6.9	178.1 ± 6.7	175.0 ± 6.5*	173.7 ± 6.7
Weight (kg)	83.8 ± 14.1	77.4 ± 13.3	82.6 ± 13.2*	86.9 ± 14.7*	88.2 ± 14.1	86.3 ± 12.9	84.7 ± 12.7
BMI (kg/m^2^)	26.6 ± 4.3	24.2 ± 3.8	25.9 ± 4.0*	27.0 ± 4.2*	27.8 ± 4.1*	28.2 ± 4.0	28.1 ± 3.7
FM (kg)	24.3 ± 9.5	19.0 ± 8.8	22.6 ± 8.8*	25.6 ± 9.5*	27.2 ± 9.1*	27.7 ± 8.4	27.9 ± 8.4
LM (kg)	56.3 ± 6.9	55.2 ± 7.2	56.9 ± 6.9*	58.1 ± 7.0*	57.8 ± 6.7	55.5 ± 6.2*	53.6 ± 5.9*
%FM	29.3 ± 7.3	24.8 ± 7.4	27.7 ± 6.9*	29.8 ± 6.5*	31.3 ± 6.2*	32.7 ± 5.7*	33.6 ± 5.9*
%LM	70.7 ± 7.3	75.2 ± 7.4	72.3 ± 6.9*	70.2 ± 6.5*	68.7 ± 6.2*	67.3 ± 5.7*	66.4 ± 5.9*
FMI (kg/m^2^)	7.7 ± 3.0	6.0 ± 2.7	7.1 ± 2.8*	8.0 ± 2.9*	8.6 ± 2.8*	9.0 ± 2.7*	9.3 ± 2.7
LMI (kg/m^2^)	17.8 ± 1.8	17.3 ± 1.9	17.8 ± 1.9*	18.1 ± 1.8*	18.2 ± 1.8	18.1 ± 1.7	17.8 ± 1.5*
Appendicular FMI (kg/m^2^)	3.1 ± 1.0	2.8 ± 1.1	3.0 ± 1.1*	3.1 ± 1.0	3.2 ± 1.0	3.2 ± 0.9	3.3 ± 0.9
Appendicular LMI (kg/m^2^)	8.3 ± 1.0	8.3 ± 1.1	8.5 ± 1.1	8.5 ± 1.0	8.5 ± 1.0	8.2 ± 1.0*	7.9 ± 0.8*
FM android/gynoid (kg)	0.7 ± 0.2	0.4 ± 0.1	0.5 ± 0.2*	0.7 ± 0.2*	0.8 ± 0.2*	0.8 ± 0.2*	0.9 ± 0.2*
FM trunk/limbs (kg)	1.4 ± 0.4	1.0 ± 0.3	1.2 ± 0.3*	1.4 ± 0.3*	1.6 ± 0.4*	1.7 ± 0.4*	1.7 ± 0.4
VAT mass (g)	1218.1 ± 939.2 (*n* = 5025)	424.6 ± 385.4 (*n* = 1188)	767.2 ± 543.2* (*n* = 869)	1243.3 ± 755.2* (*n* = 813)	1612.5 ± 894.8* (*n* = 824)	1904.0 ± 914.0* (*n* = 763)	2037.7 ± 888.0* (*n* = 568)
VAT volume (cm^3^)	1291.1 ± 995.6 (*n* = 5025)	450.1 ± 408.5 (*n* = 1188)	813.3 ± 575.8* (*n* = 869)	1317.9 ± 800.5* (*n* = 813)	1709.2 ± 948.5* (*n* = 824)	2018.2 ± 968.8* (*n* = 763)	2160.0 ± 941.3* (*n* = 568)

Women							
*n*	5747	1324	861	1040	1010	925	587
Height (cm)	164.3 ± 6.6°	165.8 ± 6.4°	165.8 ± 6.5°	165.5 ± 6.4°	164.4 ± 6.3*°	161.7 ± 6.0*°	160.4 ± 6.2°
Weight (kg)	67.5 ± 13.4°	62.1 ± 11.7°	64.8 ± 12.8*°	68.5 ± 13.5*°	70.2 ± 13.7*°	71.4 ± 13.4°	71.2 ± 13.0°
BMI (kg/m^2^)	25.1 ± 5.1°	22.6 ± 4.1°	23.6 ± 4.6*°	25.0 ± 4.9*°	26.0 ± 5.1*°	27.3 ± 5.1*°	27.7 ± 4.9
FM (kg)	25.9 ± 10.0°	21.5 ± 8.2°	23.0 ± 9.3*	25.6 ± 9.9*	28.0 ± 10.1*	30.1 ± 9.8*°	30.5 ± 9.4°
LM (kg)	39.7 ± 5.1°	38.7 ± 5.0°	39.8 ± 5.1*°	40.9 ± 5.2*°	40.3 ± 5.0°	39.5 ± 4.7°	39.0 ± 4.8°
%FM	38.3 ± 7.5°	34.9 ± 6.6°	35.5 ± 7.3°	37.4 ± 7.2*°	39.9 ± 6.9*°	42.3 ± 6.4*°	43.0 ± 6.3°
%LM	61.7 ± 7.5°	65.1 ± 6.6°	64.5 ± 7.3°	62.6 ± 7.2*°	60.1 ± 6.9*°	57.7 ± 6.4*°	57.0 ± 6.3°
FMI (kg/m^2^)	9.7 ± 3.8°	7.8 ± 3.0°	8.4 ± 3.4*°	9.4 ± 3.7*°	10.4 ± 3.8*°	11.6 ± 3.8*°	11.9 ± 3.7°
LMI (kg/m^2^)	14.7 ± 1.7°	14.1 ± 1.5°	14.5 ± 1.6*°	14.9 ± 1.7*°	14.9 ± 1.6°	15.1 ± 1.6*°	15.2 ± 1.6°
Appendicular FMI (kg/m^2^)	4.6 ± 1.6°	4.1 ± 1.4°	4.3 ± 1.5°	4.6 ± 1.6*°	4.8 ± 1.6*°	5.1 ± 1.6*°	5.2 ± 1.6°
Appendicular LMI (kg/m^2^)	6.6 ± 0.9°	6.4 ± 0.9°	6.5 ± 0.9*°	6.7 ± 1.0°	6.6 ± 0.9°	6.6 ± 0.9°	6.7 ± 0.9°
FM android/gynoid (kg)	0.4 ± 0.2°	0.3 ± 0.1°	0.3 ± 0.1*°	0.4 ± 0.1*°	0.5 ± 0.2*°	0.6 ± 0.2*°	0.6 ± 0.2°
FM trunk/limbs (kg)	1.0 ± 0.3°	0.8 ± 0.2°	0.9 ± 0.2*°	0.9 ± 0.3*°	1.1 ± 0.3*°	1.2 ± 0.3*°	1.2 ± 0.4°
VAT mass (g)	635.1 ± 574.3° (*n* = 5274)	235.6 ± 241.8° (*n* = 1104)	340.2 ± 312.6*° (*n* = 775)	522.8 ± 432.4*° (*n* = 975)	759.9 ± 528.4*° (*n* = 972)	1053.0 ± 628.1*° (*n* = 885)	1146.7 ± 631.3*° (*n* = 563)
VAT volume (cm^3^)	673.2 ± 608.8° (*n* = 5274)	249.7 ± 256.3° (*n* = 1104)	360.6 ± 331.4*° (*n* = 775)	554.1 ± 458.4*° (*n* = 975)	805.5 ± 560.1*° (*n* = 972)	1116.2 ± 665.8*° (*n* = 885)	1215.5 ± 669.1*° (*n* = 563)

It shows means ± SD.

*n* = sample size for each age group, for VAT parameters sample sizes are displayed underneath means.

*yrs* years, *BMI* body mass index, *FM* fat mass, *LM* lean mass, *FMI* fat mass/height^2^, *LMI* lean mass/height^2^, *appendicular* sum of four limbs, *VAT* visceral adipose tissue.

*p < 0.01: significant age effects on mean vs. previous age group (*T*-test for independent samples and inhomogeneous variances, Holm correction applied, Scheffe test for independent samples and homogenous variances, Wilcoxon-rank-test for independent samples which are not normally distributed).

°*p* < 0.01: significant gender effects on mean (*T*-test for independent samples, normally distributed, Wilcoxon-rank-test for not-normally distributed parameters).

### Sex differences

After stratification for sex, all body composition parameters showed differences (*p* < 0.01) in every age group, except for BMI in the oldest age group (70 to <82 years) and FM in the age groups from 30 until <60 years. Anthropometric characteristics (height, weight, and BMI) and FM parameters indicating central fat accumulation (FM trunk/limbs, FM android/gynoid, and VAT mass and volume) were higher in men, while other FM parameters (%FM, FMI, appendicular FMI in all age groups, and FM in age groups 18 to <30, 60 to <70, 70 to <82, and 18 to <82 years) were higher in women. LM parameters (LM, %LM, LMI, and appendicular LMI) were higher in men compared with women independent of age groups.

### Age differences

In women, no age differences were found in any parameter between the two oldest age groups (60 to <70 years and 70 to <82 years), except for VAT mass and volume, which increased across all age groups in both sexes.

### Age differences FM parameters

FM, FMI, and FM ratios android/gynoid and trunk/limbs increased with age groups in women from 18 to <70 years, whereas %FM and appendicular FMI started to increase later (from the age of 30 to <40 years). In men, appendicular FMI increased only between the two youngest age groups (until 30 to <40 years), while FM increased until 50 to <60 years, and FMI as well as FM trunk/limbs until 60 to <70 years. %FM and FM android/gynoid increased further between all groups.

### Age differences LM parameters

%LM decreased from the age group 30 to <40 years until the age group of 60 to <70 years in women and even further in men (from the youngest age group until the oldest age group). In men, appendicular LMI decreased in older age (from 50 to <60 years to 70 to <82 years), while LMI decreased only in the oldest age group, but increased from the youngest age group until 40 to <50 years. In women, appendicular LMI increased only in young women (between age groups 18 to <30 years and 30 to <40 years), while LMI increased until 60 to <70 years, apart from no increase between age group 40 to <50 years and 50 to <60 years.

### LMS models

**Fig. 1:**
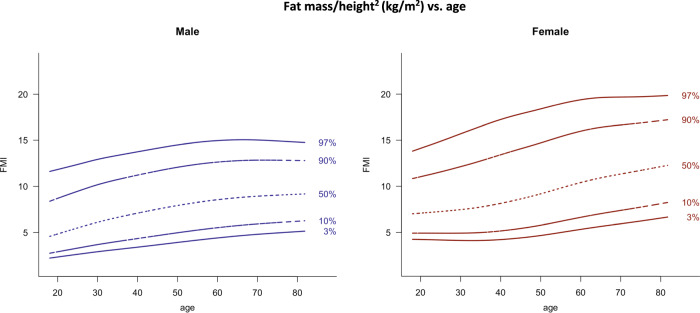
Fat mass/height^2^ (kg/m^2^) vs. age. Lines indicate 3rd, 10th, 50th, 90th, and 97th percentile. Age in years, FMI in kg/m^2^.

**Fig. 2:**
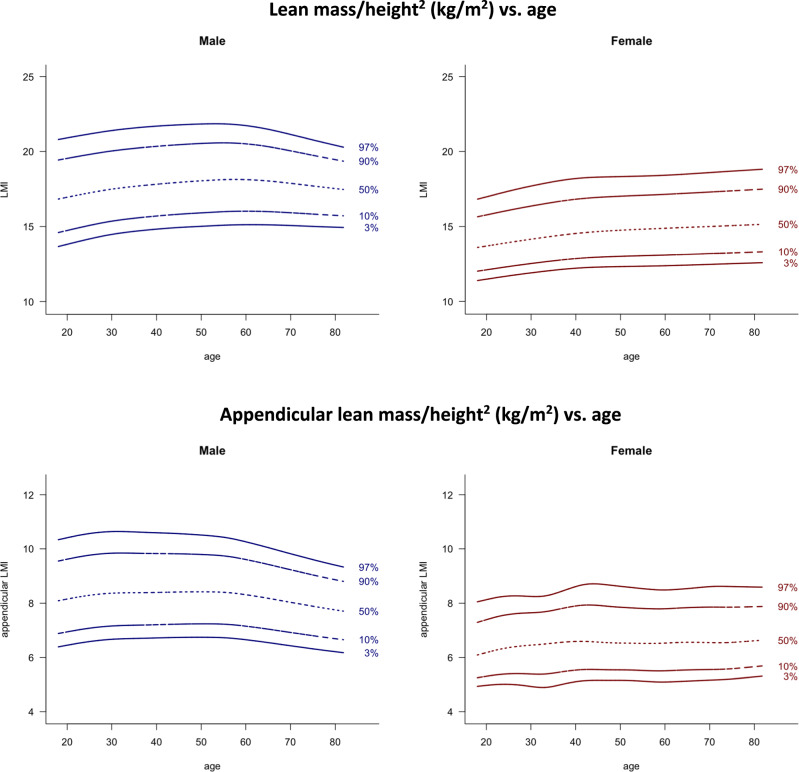
Lean mass/height^2^ (kg/m^2^) vs. age and appendicular lean mass/height^2^ (kg/m^2^) vs. age. Lines indicate 3rd, 10th, 50th, 90th, and 97th percentile. Age in years, LMI and appendicular LMI in kg/m^2^.

**Fig. 3:**
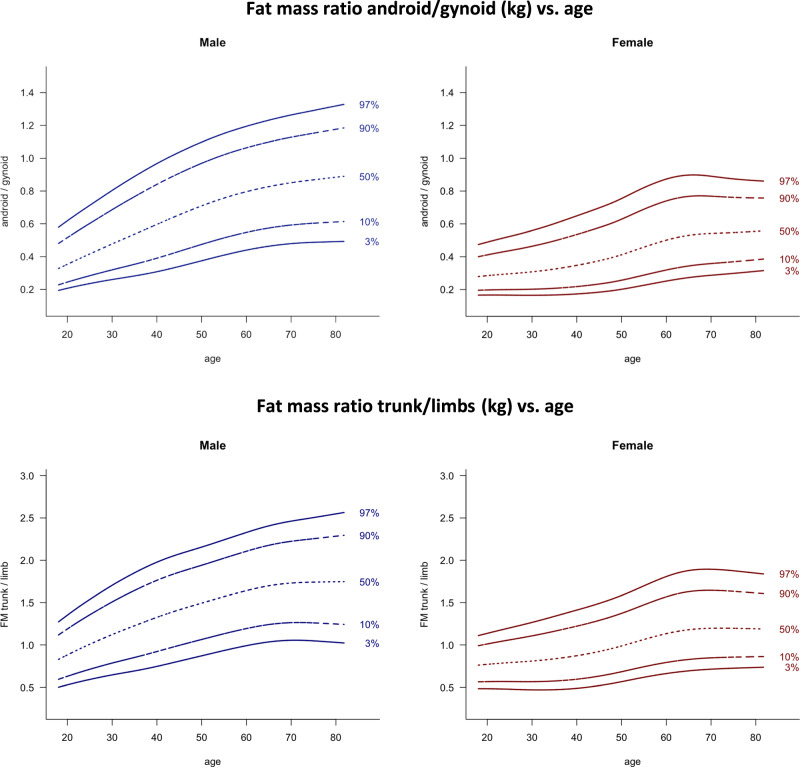
Fat mass ratio android/gynoid (kg) and fat mass ratio trunk/limbs (kg) vs. age. Lines indicate 3rd, 10th, 50th, 90th, and 97th percentile. Age in years, FM in kg.

**Fig. 4:**
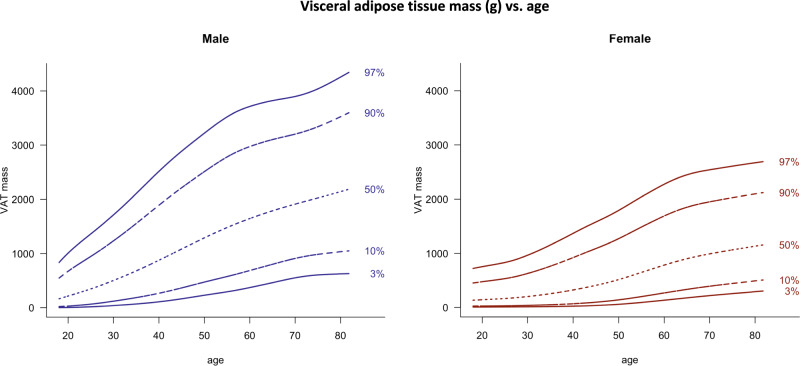
Visceral adipose tissue mass (g) vs. age. Lines indicate 3rd, 10th, 50th, 90th, and 97th percentile. Age in years, VAT mass in g.

**Fig. 5:**
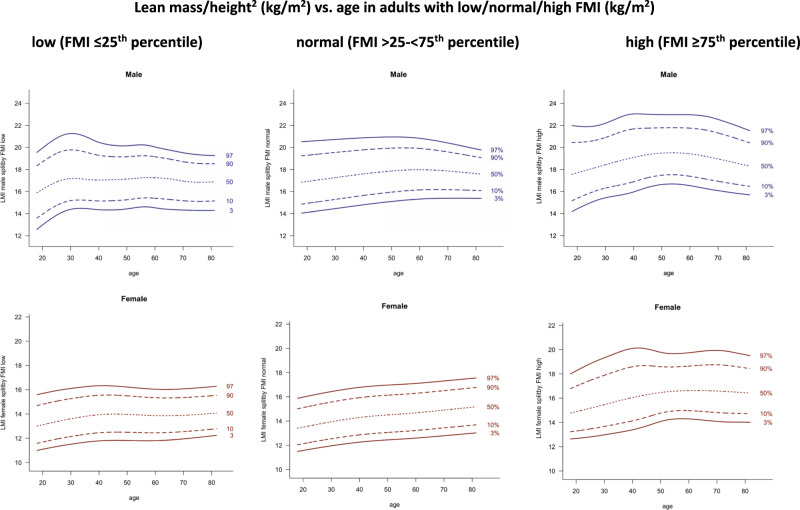
Lean mass/height^2^ (kg/m^2^) vs. age in adults with low/normal/high FMI (kg/m^2^). **a** Low FMI (≤25th percentile). **b** Normal FMI (>25 to <75th percentile). **c** High FMI (≥75th percentile). Lines indicate 3rd, 10th, 50th, 90th, and 97th percentile. Age in years, FMI and LMI in kg/m^2^.

Curves of the 3rd, 10th, 50th, 90th, and 97th percentile constructed with the LMS method for the body composition parameters FMI (Fig. 1), LMI and appendicular LMI (Fig. 2), FM android/gynoid and FM trunk/limbs (Fig. 3), and for VAT mass (Fig. 4), are shown separated by sex. In Fig. 5, LMI percentile curves of different FMI subgroups (low, normal, and high FMI) are displayed. Figures S1 and S2 show LMI and appendicular LMI percentile curves for adults in different BMI categories. Reference values for each percentile are provided in the Supplementary material (Tables S1–22).

### Comparison between LMI subgroups of different FMI categories

LMI differed (*p* < 0.01) between each FMI subgroup (low vs. normal, low vs. high, and normal vs. high) in all age groups in both sexes. Means of LMI increased by the FMI subgroups.

### Comparison between LMI and appendicular LMI of different BMI categories

LMI and appendicular LMI were different (*p* < 0.01) between each BMI category. Means of both LM parameters increased by BMI category.

## Discussion

The present study shows based on a 10.894 large European population-based cohort aged 18–81 years, age- and sex-related reference values for total and regional body composition parameters and VAT, obtained by Lunar Prodigy DXA scans. In addition, this study firstly reports LMI reference values with regard to different FM quantities, showing a positive association with increasing FMI percentiles. Moreover, LMI and appendicular LMI reference values are provided for different BMI categories.

It is well established that reference values should take age, sex, and ethnicity into account [17], and should be population- and technique-specific [18]. In addition, reference values are only applicable to the same DXA device and software they have been measured with [18]. So far, available large population-based reference values are either carried out in the United States [23–26], were measured with DXA devices from Hologic Inc. (Bedford, MA, USA) [23, 27] and iDXA [28], or show only descriptive percentile values [29]. So far, the recommended reference values of body composition parameters are those based on the American NHANES cohort [30]. Whether those reference values are applicable to populations outside America is unclear [30], but comparison with the LEAD cohort, suggest that they might not be representative for European countries (see Fig. S3). Therefore, reference values for Lunar Prodigy DXA were created, based on the large general population-based LEAD cohort, which is representative for northwest and central Europe.

### FM parameters

In this analysis, significant sex differences were found in almost all parameters, which supports the need for sex-specific reference values. The present data show higher FM parameters in women compared with men, while FM indices of abdominal fat accumulation were higher in men, which is in line with other studies [23, 24, 26]. FMI increased in both sexes between all age groups until the age of 60 to <70 years, similar to the findings of Prado et al. [31]. Imboden et al. [26] found FMI increasing until 50 to <59 years, followed by a plateau and a decrease afterward, which might be a result of a smaller sample size. Mean FM android/gynoid increased in men until the oldest age group. In women, mean FM android/gynoid as well as FM trunk/limbs in both sexes, increased until the age of 60 to <70 years, all indicating an increasing fat accumulation in the abdominal region with age.

### Visceral adipose tissue

Due to the elevated risk for CVDs and mortality associated with high amounts of abdominal and especially visceral fat accumulation, population-based VAT analysis are of great clinical importance. So far, reference values of VAT have either been based on a sample of adults aged 20–30 years [32], an American population [33], or on scans of iDXA [28, 33]. The present study firstly reports VAT reference values for Lunar Prodigy scanners from young adulthood (>18 years) until elderly (<82 years) based on a large sample of 10.299 European adults. We found that VAT mass differs between sexes, with higher amounts in men and increasing values with age throughout the entire age range in men and women. A multi-centre, multi-approached study by Swainson et al. [28] found decreasing VAT values from the age of >70 years, which is in contrast to our findings. According to Swainson et al., this might be a result of the scarce data from elderly participants in their study sample. In addition, we found higher VAT values across all ages, which might also be a consequence of our larger sample size. Nevertheless, DXA machine differences have to be considered as well.

### Lean mass

In the present study, LM parameters (LM, %LM, LMI, and appendicular LMI) were higher in men across all age groups, which is in accordance with results from other cohorts [25]. Since LM and FM are related closely to each other, and weight changes affect both tissue components, obese people are supposed to have higher LM values than normal-weight subjects [17]. Hence, it is important to consider the amount of FM in the establishment of LM reference centiles. Several different approaches of addressing the relationship between FM and LM have been proposed [31, 34] to identify persons with abnormal proportions of those two body compartments, which can subsequently be related to certain phenotypes. To the authors’ best knowledge, this is the first study reporting reference values for LMI with regard to FMI quantities. The positive association is supported by our findings since mean LMI values increased with subjects of higher FMI categories. While appendicular LMI stayed the same in young and middle-aged men, it decreased from the age of 50 to <60 years, whereas an increase was found in women from 18 to <30 to 30 to <40 years, remaining unchanged afterwards. Those results support the findings of Prado et al., which showed an earlier and sharper decline of appendicular LMI in men compared with women [31]. We hypothesised that this might be an effect related to the correlated increase of appendicular FMI throughout age, which seems to play a greater and longer-persisting role in women. Another hypothesis is that the broader distribution of appendicular LMI in young men indicates a subgroup with above-average skeletal muscle mass in young age, which might disappear as a consequence of reduced physical activity associated with aging. However, with the present data, causalities cannot be drawn.

### LMI and appendicular LMI in BMI categories

Although FMI is a more precise parameter of the FM proportion, BMI is a widely used body composition parameter for defining overweight and obesity. For clinical use and to enable comparison with other study cohorts, LMI and appendicular LMI reference values were constructed after dividing the study sample according to the WHO BMI categories. In line with our findings of increased LMI values in subjects within higher FMI percentiles, LMI and appendicular LMI increased with BMI category. Our results are in alignment with the study by Prado et al. [31], who proposed BMI-specific reference percentiles for the first time and found that the association between LM parameters and age is modified by BMI.

So far, the suggested approaches, which aim to reflect the relationship between LM and FM, as well as thresholds for abnormal FMI and LMI values have not been validated yet [30]. The authors encourage that this topic should be addressed in the future to enable the identification of phenotypes at risk.

### Strengths

The major strength of the present study is the randomised, population-based recruitment of the study cohort, which is representative for northwest and central Europe [35], as well as the standardised, single-centre measurements. The second strength is the mathematical approach with the LMS model, a widely used statistical method for the construction of reference curves [36, 37], with which the dependence on age is accounted for continuously. This is preferable to the standard descriptive alternative of dividing the cohort into arbitrary age groups, which might influence results. A further advantage of this method is the possibility to calculate *z*-scores for all individuals, based on the provided *L*, *M*, and *S* values for each age and sex.

### Limitations

Even though the single-centre setting is an advantage in regard to unity, it is a limitation in terms of the population sample since the reference values are based solely on inhabitants of one single country. As the LEAD cohort is only representative for Caucasians and European inhabitants, the present reference values are not applicable to other ethnicities. The prevalence of obesity in Austria lies within the European range, as estimated by the WHO [19], indicating that these reference values might be suitable for other European countries as well. However, evaluation in other European population-based cohorts is needed to prove generalisability. To the authors’ knowledge, this is the first study providing reference values for Lunar Prodigy based on a well-sampled European general-population cohort, using the same approach as the for American populations recommended reference percentiles based on the NHANES cohort. Regarding the differences between DXA systems and devices, the body composition reference values are further only applicable to Lunar Prodigy DXA systems. Moreover, it should be noted that even though studies have shown good correlation between the VAT measurements of DXA and the gold standard methods CT and MRI [6, 9], VAT is assessed in different areas. Cheung et al. showed that DXA underestimates VAT when compared with CT, however a stronger correlation between DXA and MRI was found [9].

## Conclusion

This study provides for the first time, based on a 10.894 large European population-based cohort aged 18–81 years, age- and sex-related reference values for total and regional body composition parameters and VAT mass, for Lunar Prodigy DXA. Moreover, LMI percentile curves have been generated, taking the amount of FM into account, showing a positive association with increasing FMI percentiles. Particularly important for clinical usage, LMI and appendicular LMI reference values were constructed for different WHO BMI categories.

## Supplementary information

FigureS1

FigureS2

FigureS3

Tables S1-22

## Data Availability

The datasets used and/or analysed during the current study are available from the corresponding author on reasonable request.
