# Supplementary material for: Reference values of body composition parameters and visceral adipose tissue (VAT) by DXA in adults aged 18–81 years—results from the LEAD cohort
Source: Eur J Clin Nutr. 2020 Mar 2;74(8):1181–91. doi: 10.1038/s41430-020-0596-5 (PMC7402993; doi:10.1038/s41430-020-0596-5)

Appendicular lean mass/height^2 (kg/m^2) vs. age in adults of different BMI (kg/m^2) categories

normal (BMI 18.5-<25 kg/m^2)

overweight (BMI 25-<30 kg/m^2)

obesity (BMI ≥30 kg/m^2)

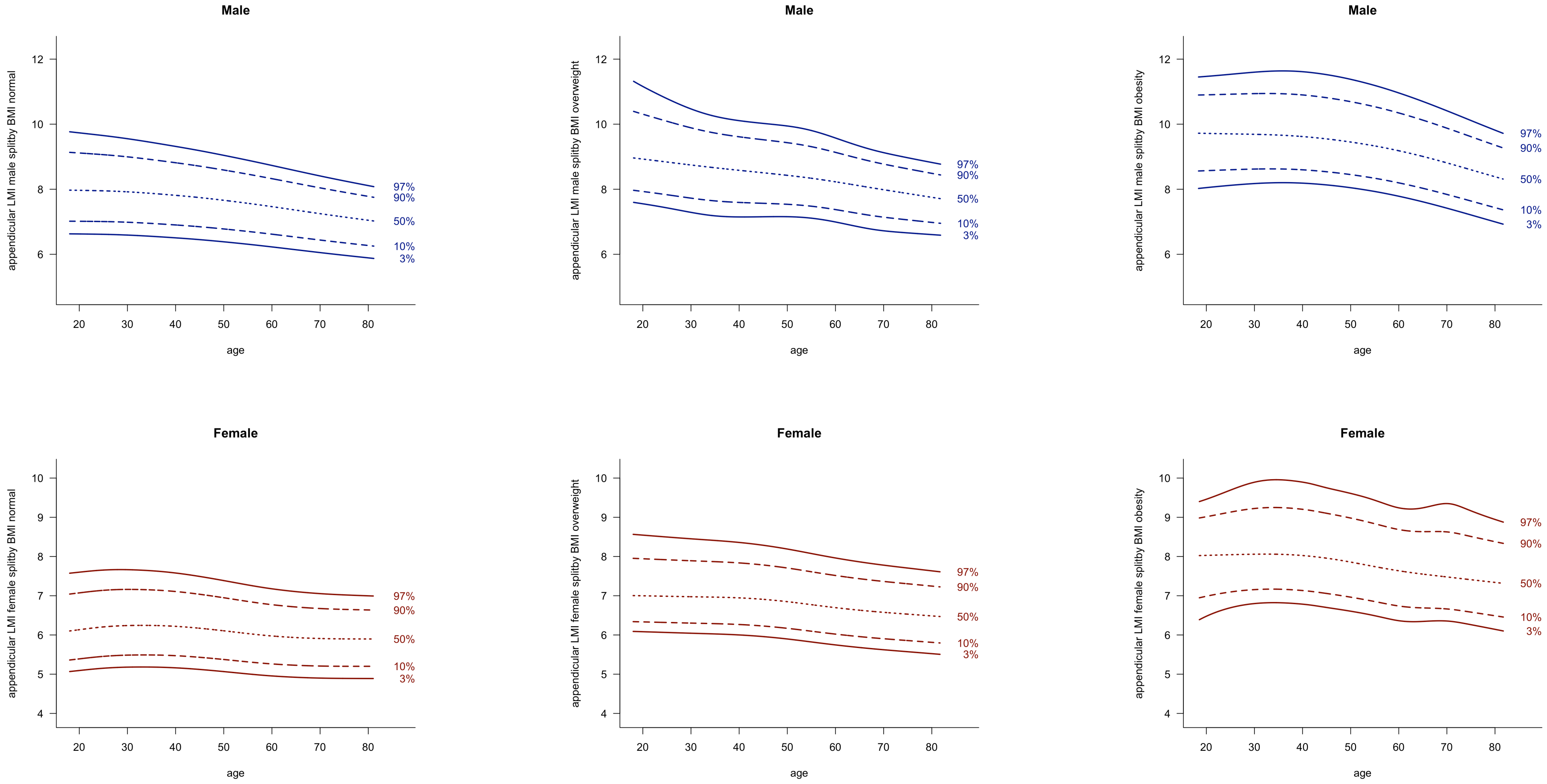

Supplement: Supplementary file 2 — FigureS2 [file 41430_2020_596_MOESM2_ESM.pdf]
