# Supplementary material for: Reference values of body composition parameters and visceral adipose tissue (VAT) by DXA in adults aged 18–81 years—results from the LEAD cohort
Source: Eur J Clin Nutr. 2020 Mar 2;74(8):1181–91. doi: 10.1038/s41430-020-0596-5 (PMC7402993; doi:10.1038/s41430-020-0596-5)
Supplement: Supplementary file 3 — FigureS3 [file 41430_2020_596_MOESM3_ESM.pdf]

Figure S1. Evaluation of the NHANES reference values in the LEAD cohort.

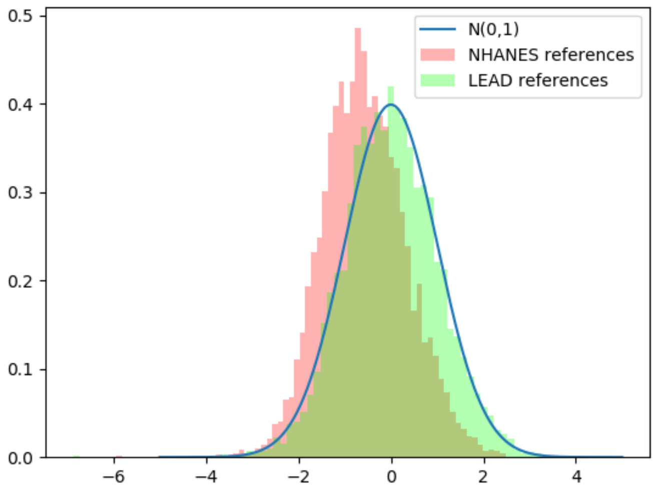

z-scores of FMI in females of the LEAD cohort

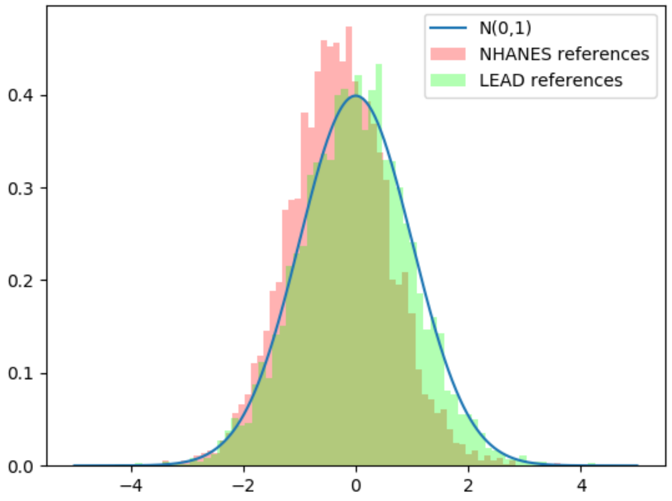

z-scores of LMI in males of the LEAD cohort

Legend: This figure shows with two examples (FMI in females, LMI in males), that z-scores of the LEAD cohort calculated with the NHANES L, M, S values (rose) do not fit well, as these distributions have smaller standard deviations and are shifted to the left. For the evaluation we used the sex- and age-specific parameters applicable to Lunar Prodigy systems for white adults (Fan et. al, J Clin Densitom., 2014). In contrast, z-scores calculated with the L, M, S values published in this article (green) correspond to a standard Gaussian distribution (blue curve). This was confirmed by Kolmogorov-Smirnov test ($p<0.05$, using multiple testing correction) for all parameters evaluated (FMI, LMI, appendicular LMI, FM trunk/limb).
