# Supplementary material for: Reference values of body composition parameters and visceral adipose tissue (VAT) by DXA in adults aged 18–81 years—results from the LEAD cohort
Source: Eur J Clin Nutr. 2020 Mar 2;74(8):1181–91. doi: 10.1038/s41430-020-0596-5 (PMC7402993; doi:10.1038/s41430-020-0596-5)
Supplement: Supplementary file 4 — Tables S1-22 [file 41430_2020_596_MOESM4_ESM.pdf]

Tables S1-22: LMS parameter modeled percentiles (3rd, 10th, 50th, 90th, 97th) of different body composition parameters (FMI, LMI, appendicular LMI, FM android/gynoid, FM trunk/limb, VAT mass, LMI by FMI categories, LMI and appendicular LMI by BMI categories)

S1: LMS parameter modeled percentiles of fat mass/height² (kg/m²) vs. age in males.

**FMI males** (n=5147) **age (yrs)**	**L**	**M**	**S**	**3rd**	**10th**	**50th**	**90th**	**97th**
18	-0,309	4,580	0,430	2,224	2,752	4,580	8,382	11,620
20	-0,256	4,845	0,424	2,344	2,912	4,845	8,703	11,833
25	-0,128	5,498	0,410	2,636	3,307	5,498	9,470	12,379
30	-0,013	6,118	0,396	2,916	3,690	6,118	10,179	12,929
35	0,081	6,642	0,382	3,166	4,028	6,642	10,739	13,360
40	0,153	7,106	0,369	3,409	4,348	7,106	11,223	13,751
45	0,204	7,539	0,357	3,663	4,665	7,539	11,671	14,138
50	0,239	7,934	0,345	3,924	4,975	7,934	12,073	14,494
55	0,261	8,274	0,333	4,177	5,264	8,274	12,398	14,775
60	0,277	8,553	0,321	4,413	5,523	8,553	12,635	14,960
65	0,291	8,773	0,311	4,625	5,747	8,773	12,784	15,045
70	0,310	8,932	0,300	4,805	5,932	8,932	12,843	15,022
75	0,335	9,046	0,290	4,956	6,085	9,046	12,836	14,921
80	0,364	9,142	0,280	5,097	6,225	9,142	12,807	14,797
81	0,370	9,161	0,278	5,125	6,253	9,161	12,801	14,773

S2: LMS parameter modeled percentiles of fat mass/height² (kg/m²) vs. age in females.

**FMI females** (n=5747) **age (yrs)**	**L**	**M**	**S**	**3rd**	**10th**	**50th**	**90th**	**97th**
18	-0,521	7,012	0,304	4,253	4,920	7,012	10,833	13,812
20	-0,491	7,082	0,311	4,235	4,921	7,082	11,036	14,114
25	-0,415	7,259	0,330	4,178	4,916	7,259	11,560	14,885
30	-0,340	7,463	0,349	4,125	4,921	7,463	12,126	15,691
35	-0,264	7,741	0,364	4,123	4,984	7,741	12,743	16,489
40	-0,189	8,141	0,372	4,221	5,158	8,141	13,415	17,239
45	-0,114	8,623	0,371	4,404	5,425	8,623	14,062	17,848
50	-0,038	9,176	0,365	4,662	5,774	9,176	14,706	18,389
55	0,037	9,800	0,355	4,987	6,196	9,800	15,382	18,943
60	0,112	10,403	0,343	5,327	6,630	10,403	15,973	19,385
65	0,188	10,913	0,330	5,643	7,026	10,913	16,391	19,625
70	0,263	11,326	0,316	5,932	7,379	11,326	16,646	19,681
75	0,338	11,719	0,303	6,228	7,734	11,719	16,871	19,720
80	0,414	12,127	0,289	6,546	8,108	12,127	17,122	19,810
81	0,429	12,208	0,287	6,611	8,185	12,208	17,173	19,831

S3: LMS parameter modeled percentiles of lean mass/height^2 (kg/m^2) vs. age in males.

**LMI males** (n=5147) **age (yrs)**	**L**	**M**	**S**	**3rd**	**10th**	**50th**	**90th**	**97th**
18	-0,071	16,828	0,112	13,662	14,595	16,828	19,430	20,792
20	-0,114	16,950	0,110	13,812	14,735	16,950	19,541	20,902
25	-0,218	17,243	0,107	14,172	15,073	17,243	19,806	21,163
30	-0,308	17,490	0,104	14,471	15,353	17,490	20,034	21,389
35	-0,355	17,671	0,102	14,675	15,549	17,671	20,205	21,560
40	-0,343	17,817	0,101	14,822	15,698	17,817	20,339	21,683
45	-0,275	17,942	0,100	14,933	15,817	17,942	20,446	21,769
50	-0,177	18,045	0,099	15,012	15,908	18,045	20,529	21,827
55	-0,079	18,123	0,098	15,084	15,987	18,123	20,569	21,833
60	0,009	18,123	0,096	15,119	16,018	18,123	20,502	21,718
65	0,080	18,034	0,093	15,110	15,990	18,034	20,316	21,471
70	0,143	17,877	0,090	15,064	15,916	17,877	20,042	21,129
75	0,212	17,701	0,086	15,006	15,827	17,701	19,745	20,763
80	0,284	17,528	0,083	14,953	15,742	17,528	19,456	20,407
81	0,298	17,495	0,082	14,942	15,725	17,495	19,400	20,338

S4: LMS parameter modeled percentiles of lean mass/height^2 (kg/m^2) vs. age in females.

**LMI females** (n=5747) **age (yrs)**	**L**	**M**	**S**	**3rd**	**10th**	**50th**	**90th**	**97th**
18	-0,928	13,602	0,103	11,391	12,016	13,602	15,649	16,824
20	-1,004	13,698	0,103	11,482	12,105	13,698	15,774	16,977
25	-1,182	13,932	0,103	11,703	12,324	13,932	16,080	17,358
30	-1,293	14,152	0,103	11,901	12,525	14,152	16,361	17,697
35	-1,333	14,358	0,103	12,076	12,707	14,358	16,614	17,987
40	-1,287	14,534	0,104	12,212	12,855	14,534	16,817	18,198
45	-1,126	14,663	0,104	12,286	12,950	14,663	16,942	18,290
50	-0,945	14,752	0,104	12,322	13,007	14,752	17,018	18,327
55	-0,813	14,820	0,105	12,347	13,049	14,820	17,080	18,363
60	-0,734	14,881	0,105	12,377	13,091	14,881	17,143	18,416
65	-0,725	14,942	0,105	12,420	13,139	14,942	17,219	18,499
70	-0,755	15,000	0,105	12,469	13,188	15,000	17,298	18,595
75	-0,778	15,059	0,106	12,517	13,239	15,059	17,378	18,691
80	-0,780	15,123	0,106	12,564	13,291	15,123	17,459	18,783
81	-0,780	15,136	0,106	12,574	13,301	15,136	17,475	18,801

S5: LMS parameter modeled percentiles of appendicular lean mass/height^2 (kg/m^2) vs. age in males.

appendicular LMI males (n=5147) age (yrs)	L	M	S	3rd	10th	50th	90th	97th
18	-0,161	8,090	0,128	6,392	6,884	8,090	9,549	10,335
20	-0,178	8,154	0,127	6,453	6,945	8,154	9,618	10,408
25	-0,215	8,291	0,125	6,587	7,079	8,291	9,765	10,563
30	-0,236	8,368	0,124	6,670	7,161	8,368	9,838	10,635
35	-0,228	8,387	0,122	6,702	7,189	8,387	9,838	10,623
40	-0,186	8,397	0,121	6,721	7,208	8,397	9,825	10,591
45	-0,106	8,413	0,119	6,740	7,229	8,413	9,816	10,558
50	0,005	8,420	0,118	6,745	7,239	8,420	9,792	10,508
55	0,130	8,402	0,116	6,729	7,228	8,402	9,740	10,426
60	0,260	8,313	0,115	6,655	7,154	8,313	9,606	10,259
65	0,379	8,182	0,113	6,549	7,045	8,182	9,426	10,046
70	0,482	8,034	0,112	6,433	6,923	8,034	9,231	9,820
75	0,580	7,889	0,111	6,320	6,804	7,889	9,041	9,603
80	0,677	7,754	0,109	6,214	6,693	7,754	8,864	9,400
81	0,696	7,729	0,109	6,195	6,672	7,729	8,831	9,361

S6: LMS parameter modeled percentiles of appendicular lean mass/height^2 (kg/m^2) vs. age in females.

appendicular LMI females (n=5747) age (yrs)	L	M	S	3rd	10th	50th	90th	97th
18	-1,14	6,093	0,127	4,934	5,249	6,093	7,293	8,049
20	-0,926	6,182	0,129	4,970	5,303	6,182	7,392	8,128
25	-0,342	6,364	0,133	5,008	5,394	6,364	7,584	8,263
30	0,370	6,455	0,136	4,929	5,388	6,455	7,647	8,249
35	0,520	6,530	0,139	4,934	5,419	6,530	7,739	8,338
40	-0,170	6,591	0,139	5,103	5,530	6,591	7,897	8,611
45	-0,584	6,569	0,138	5,156	5,550	6,569	7,919	8,709
50	-0,618	6,533	0,136	5,155	5,539	6,533	7,851	8,622
55	-0,434	6,52	0,135	5,122	5,517	6,520	7,807	8,536
60	-0,206	6,531	0,136	5,093	5,505	6,531	7,795	8,487
65	-0,249	6,561	0,136	5,124	5,535	6,561	7,836	8,538
70	-0,541	6,550	0,135	5,161	5,550	6,550	7,857	8,611
75	-0,659	6,558	0,133	5,204	5,581	6,558	7,855	8,615
80	-0,614	6,617	0,129	5,280	5,655	6,617	7,872	8,597
81	-0,602	6,629	0,128	5,297	5,671	6,629	7,877	8,594

S7: LMS parameter modeled percentiles of fat mass ratio android/gynoid (kg) vs. age in males.

**FM android/gynoid males** (n=5147) **age (yrs)**	**L**	**M**	**S**	**3rd**	**10th**	**50th**	**90th**	**97th**
18	-0,154	0,328	0,290	0,194	0,228	0,328	0,480	0,579
20	-0,081	0,354	0,291	0,207	0,245	0,354	0,516	0,619
25	0,101	0,416	0,294	0,236	0,284	0,416	0,603	0,713
30	0,273	0,478	0,297	0,260	0,319	0,478	0,686	0,804
35	0,426	0,537	0,299	0,282	0,353	0,537	0,766	0,889
40	0,550	0,596	0,295	0,308	0,391	0,596	0,841	0,967
45	0,645	0,655	0,284	0,340	0,432	0,655	0,908	1,037
50	0,712	0,709	0,273	0,375	0,475	0,709	0,969	1,098
55	0,752	0,757	0,262	0,409	0,514	0,757	1,021	1,151
60	0,769	0,796	0,253	0,440	0,548	0,796	1,063	1,194
65	0,772	0,827	0,248	0,464	0,574	0,827	1,099	1,232
70	0,773	0,851	0,246	0,479	0,592	0,851	1,128	1,264
75	0,779	0,868	0,247	0,488	0,604	0,868	1,152	1,291
80	0,788	0,884	0,250	0,492	0,611	0,884	1,176	1,319
81	0,790	0,887	0,250	0,492	0,613	0,887	1,181	1,324

S8: LMS parameter modeled percentiles of fat mass ratio android/gynoid (kg) vs. age in females.

**FM android/gynoid females** (n=5747) **age (yrs)**	**L**	**M**	**S**	**3rd**	**10th**	**50th**	**90th**	**97th**
18	-0,033	0,279	0,279	0,166	0,196	0,279	0,400	0,474
20	-0,015	0,285	0,287	0,166	0,197	0,285	0,411	0,489
25	0,030	0,296	0,306	0,166	0,200	0,296	0,437	0,524
30	0,076	0,308	0,324	0,165	0,202	0,308	0,464	0,559
35	0,121	0,325	0,340	0,167	0,208	0,325	0,497	0,601
40	0,166	0,347	0,350	0,173	0,218	0,347	0,535	0,649
45	0,211	0,375	0,352	0,184	0,234	0,375	0,578	0,698
50	0,256	0,412	0,348	0,201	0,257	0,412	0,629	0,755
55	0,302	0,458	0,339	0,226	0,288	0,458	0,689	0,821
60	0,347	0,501	0,326	0,252	0,319	0,501	0,740	0,873
65	0,392	0,530	0,311	0,273	0,344	0,530	0,768	0,898
70	0,437	0,542	0,295	0,287	0,358	0,542	0,769	0,891
75	0,482	0,547	0,279	0,299	0,369	0,547	0,762	0,874
80	0,528	0,555	0,265	0,311	0,381	0,555	0,758	0,864
81	0,537	0,556	0,262	0,314	0,384	0,556	0,758	0,862

S9: LMS parameter modeled percentiles of fat mass ratio trunk/limb (kg) vs. age in males.

**FM trunk/limb males** (n=5147) **age (yrs)**	**L**	**M**	**S**	**3rd**	**10th**	**50th**	**90th**	**97th**
18	0,351	0,829	0,246	0,501	0,594	0,829	1,118	1,272
20	0,389	0,882	0,247	0,529	0,630	0,882	1,189	1,352
25	0,485	1,008	0,249	0,592	0,712	1,008	1,356	1,537
30	0,581	1,123	0,251	0,646	0,786	1,123	1,508	1,704
35	0,674	1,227	0,252	0,693	0,852	1,227	1,644	1,852
40	0,755	1,326	0,248	0,746	0,922	1,326	1,763	1,977
45	0,819	1,415	0,239	0,808	0,995	1,415	1,859	2,074
50	0,861	1,494	0,229	0,871	1,065	1,494	1,941	2,156
55	0,876	1,571	0,222	0,934	1,132	1,571	2,025	2,242
60	0,868	1,642	0,217	0,991	1,194	1,642	2,106	2,329
65	0,842	1,699	0,215	1,035	1,241	1,699	2,177	2,406
70	0,809	1,731	0,217	1,055	1,263	1,731	2,224	2,462
75	0,775	1,741	0,223	1,048	1,260	1,741	2,255	2,504
80	0,742	1,746	0,232	1,030	1,248	1,746	2,284	2,548
81	0,736	1,747	0,234	1,026	1,245	1,747	2,290	2,557

S10: LMS parameter modeled percentiles of fat mass ratio trunk/limb (kg) vs. age in females.

**FM trunk/limb females** (n=5747) **age (yrs)**	**L**	**M**	**S**	**3rd**	**10th**	**50th**	**90th**	**97th**
18	0,439	0,761	0,219	0,483	0,564	0,761	0,991	1,111
20	0,432	0,771	0,225	0,483	0,566	0,771	1,012	1,137
25	0,413	0,791	0,242	0,477	0,567	0,791	1,060	1,202
30	0,395	0,810	0,260	0,470	0,566	0,810	1,107	1,266
35	0,377	0,836	0,274	0,472	0,574	0,836	1,162	1,338
40	0,358	0,873	0,279	0,488	0,595	0,873	1,222	1,412
45	0,340	0,922	0,277	0,520	0,632	0,922	1,290	1,491
50	0,321	0,988	0,271	0,566	0,683	0,988	1,373	1,584
55	0,303	1,064	0,267	0,618	0,742	1,064	1,473	1,698
60	0,284	1,134	0,265	0,662	0,793	1,134	1,568	1,808
65	0,266	1,180	0,263	0,694	0,828	1,180	1,630	1,878
70	0,248	1,197	0,258	0,713	0,847	1,197	1,645	1,894
75	0,229	1,197	0,252	0,725	0,856	1,197	1,634	1,876
80	0,211	1,191	0,245	0,734	0,861	1,191	1,614	1,848
81	0,207	1,190	0,243	0,735	0,862	1,190	1,610	1,842

S11: LMS parameter modeled percentiles of visceral adipose tissue mass (g) vs. age in males.

**VAT mass males** (n=5025) **age (yrs)**	**L**	**M**	**S**	**3rd**	**10th**	**50th**	**90th**	**97th**
18	0,352	164,402	1,163	2,522	19,875	164,402	544,54	831,752
20	0,362	215,556	1,099	4,797	30,106	215,556	672,766	1008,762
25	0,385	348,315	0,955	16,397	66,532	348,315	949,692	1364,701
30	0,408	504,977	0,844	39,158	121,24	504,977	1236,749	1716,580
35	0,431	682,589	0,773	69,347	187,509	682,589	1557,064	2109,543
40	0,455	879,984	0,716	109,322	268,184	879,984	1895,154	2516,858
45	0,478	1088,381	0,661	164,735	367,266	1088,381	2216,959	2888,344
50	0,501	1294,367	0,614	230,27	475,575	1294,367	2513,252	3220,267
55	0,525	1485,814	0,580	293,922	578,783	1485,814	2784,187	3522,000
60	0,548	1652,621	0,542	372,245	690,048	1652,621	2976,178	3712,972
65	0,571	1796,054	0,502	463,077	806,022	1796,054	3105,718	3820,557
70	0,594	1920,537	0,468	552,969	915,254	1920,537	3207,081	3897,333
75	0,618	2035,508	0,453	606,551	991,216	2035,508	3344,046	4037,753
80	0,641	2152,572	0,454	624,503	1038,78	2152,572	3530,371	4254,737
81	0,646	2176,451	0,455	626,538	1047,223	2176,451	3570,099	4301,663

S12: LMS parameter modeled percentiles of visceral adipose tissue mass (g) vs. age in females.

**VAT mass females** (n=5274) **age (yrs)**	**L**	**M**	**S**	**3rd**	**10th**	**50th**	**90th**	**97th**
18	0,242	137,633	1,090	8,081	24,993	137,633	458,575	727,408
20	0,248	146,029	1,081	8,631	26,746	146,029	480,756	758,358
25	0,261	167,825	1,057	10,183	31,552	167,825	535,452	832,810
30	0,274	203,256	1,032	12,722	39,302	203,256	628,655	964,506
35	0,287	255,221	1,002	16,906	51,380	255,221	761,847	1151,824
40	0,300	322,79	0,959	24,015	69,616	322,79	919,023	1364,766
45	0,313	407,497	0,895	37,181	98,279	407,497	1086,076	1575,645
50	0,327	512,956	0,821	59,856	141,362	512,956	1267,110	1790,654
55	0,340	644,199	0,751	94,190	200,976	644,199	1480,850	2041,105
60	0,353	778,692	0,693	135,833	268,249	778,692	1686,238	2275,688
65	0,366	895,594	0,647	179,037	333,536	895,594	1847,131	2449,518
70	0,379	984,534	0,607	220,43	391,518	984,534	1947,015	2542,807
75	0,392	1057,257	0,577	257,291	441,623	1057,257	2023,259	2610,289
80	0,406	1123,21	0,553	291,383	487,490	1123,210	2093,114	2673,336
81	0,408	1135,92	0,548	298,161	496,502	1135,920	2106,354	2685,180

S13: LMS parameter modeled percentiles of lean mass/height^2 (kg/m^2) vs. age in males with low fat mass/height^2 (kg/m^2).

**LMI males with low FMI** (n=1277) **age (yrs)**	**L**	**M**	**S**	**3rd**	**10th**	**50th**	**90th**	**97th**
18	0,576	15,893	0,117	12,572	13,594	15,893	18,343	19,539
20	0,303	16,196	0,114	12,971	13,944	16,196	18,688	19,941
25	-0,340	16,875	0,108	13,858	14,735	16,875	19,453	20,841
30	-0,930	17,183	0,103	14,384	15,175	17,183	19,776	21,265
35	-1,035	17,125	0,098	14,465	15,216	17,125	19,595	21,016
40	-0,354	17,044	0,094	14,354	15,144	17,044	19,281	20,465
45	0,269	17,079	0,091	14,331	15,169	17,079	19,159	20,192
50	0,348	17,131	0,089	14,425	15,254	17,131	19,152	20,147
55	0,241	17,240	0,087	14,596	15,403	17,240	19,240	20,233
60	0,886	17,258	0,085	14,527	15,391	17,258	19,149	20,040
65	1,390	17,156	0,083	14,383	15,287	17,156	18,948	19,763
70	1,151	16,946	0,081	14,321	15,164	16,946	18,70	19,512
75	1,161	16,843	0,080	14,283	15,106	16,843	18,552	19,342
80	1,508	16,871	0,078	14,285	15,133	16,871	18,522	19,268
81	1,578	16,883	0,078	14,291	15,144	16,883	18,523	19,262

S14: P LMS parameter modeled percentiles of lean mass/height^2 (kg/m^2) vs. age in males with normal fat mass/height^2 (kg/m^2).

**LMI males with normal FMI** (n=2593) **age (yrs)**	**L**	**M**	**S**	**3rd**	**10th**	**50th**	**90th**	**97th**
18	-0,430	16,846	0,101	14,045	14,860	16,846	19,234	20,519
20	-0,382	16,916	0,100	14,112	14,931	16,916	19,286	20,553
25	-0,263	17,092	0,098	14,281	15,108	17,092	19,416	20,639
30	-0,143	17,266	0,096	14,452	15,287	17,266	19,542	20,723
35	-0,023	17,437	0,094	14,626	15,468	17,437	19,664	20,804
40	0,096	17,601	0,091	14,798	15,644	17,601	19,777	20,875
45	0,216	17,745	0,089	14,956	15,804	17,745	19,868	20,927
50	0,336	17,865	0,087	15,097	15,946	17,865	19,933	20,951
55	0,455	17,954	0,084	15,224	16,067	17,954	19,955	20,931
60	0,575	17,984	0,081	15,318	16,148	17,984	19,904	20,829
65	0,695	17,949	0,078	15,371	16,179	17,949	19,774	20,645
70	0,814	17,864	0,075	15,391	16,171	17,864	19,587	20,403
75	0,934	17,752	0,071	15,391	16,141	17,752	19,373	20,134
80	1,054	17,630	0,068	15,382	16,100	17,630	19,153	19,863
81	1,078	17,606	0,067	15,380	16,092	17,606	19,109	19,810

S15: LMS parameter modeled percentiles of lean mass/height^2 (kg/m^2) vs. age in males with high fat mass/height^2 (kg/m^2).

**LMI males with high FMI** (n=1277) **age (yrs)**	**L**	**M**	**S**	**3rd**	**10th**	**50th**	**90th**	**97th**
18	-0,219	17,558	0,117	14,170	15,155	17,558	20,441	21,987
20	-0,223	17,702	0,111	14,430	15,385	17,702	20,460	21,929
25	-0,231	18,064	0,100	15,020	15,916	18,064	20,580	21,904
30	-0,239	18,435	0,097	15,432	16,318	18,435	20,902	22,195
35	-0,247	18,787	0,099	15,664	16,583	18,787	21,367	22,725
40	-0,255	19,098	0,097	15,966	16,889	19,098	21,682	23,040
45	-0,263	19,339	0,090	16,378	17,255	19,339	21,751	23,010
50	-0,271	19,480	0,086	16,633	17,479	19,480	21,782	22,977
55	-0,279	19,505	0,085	16,677	17,518	19,505	21,790	22,976
60	-0,287	19,411	0,087	16,533	17,387	19,411	21,749	22,967
65	-0,295	19,218	0,090	16,299	17,162	19,218	21,605	22,853
70	-0,303	18,957	0,090	16,081	16,932	18,957	21,310	22,541
75	-0,311	18,668	0,088	15,891	16,714	18,668	20,934	22,118
80	-0,319	18,379	0,085	15,736	16,520	18,379	20,524	21,640
81	-0,321	18,321	0,084	15,708	16,484	18,321	20,440	21,541

S16: LMS parameter modeled percentiles of lean mass/height^2 (kg/m^2) vs. age in females with low fat mass/height^2 (kg/m^2).

**LMI females with low FMI** (n=1452) **age (yrs)**	**L**	**M**	**S**	**3rd**	**10th**	**50th**	**90th**	**97th**
18	-0,477	13,004	0,093	11,000	11,586	13,004	14,694	15,597
20	-0,413	13,116	0,092	11,096	11,689	13,116	14,802	15,696
25	-0,247	13,384	0,091	11,326	11,936	13,384	15,058	15,931
30	-0,049	13,609	0,089	11,515	12,142	13,609	15,263	16,107
35	0,169	13,804	0,088	11,674	12,321	13,804	15,433	16,247
40	0,389	13,947	0,086	11,790	12,453	13,947	15,546	16,330
45	0,592	13,996	0,085	11,828	12,503	13,996	15,557	16,310
50	0,715	13,974	0,084	11,821	12,496	13,974	15,498	16,226
55	0,734	13,917	0,083	11,802	12,466	13,917	15,410	16,121
60	0,656	13,869	0,081	11,805	12,450	13,869	15,339	16,044
65	0,489	13,863	0,080	11,857	12,478	13,863	15,322	16,030
70	0,259	13,897	0,079	11,948	12,546	13,897	15,354	16,072
75	0,011	13,963	0,078	12,066	12,641	13,963	15,422	16,155
80	-0,233	14,044	0,076	12,194	12,749	14,044	15,506	16,254
81	-0,282	14,062	0,076	12,220	12,771	14,062	15,524	16,275

S17: LMS parameter modeled percentiles of lean mass/height^2 (kg/m^2) vs. age in females with normal fat mass/height^2 (kg/m^2).

**LMI females with normal FMI** (n=2860) **age (yrs)**	**L**	**M**	**S**	**3rd**	**10th**	**50th**	**90th**	**97th**
18	-0,631	13,398	0,086	11,493	12,050	13,398	15,010	15,876
20	-0,602	13,493	0,085	11,576	12,136	13,493	15,110	15,975
25	-0,529	13,726	0,085	11,776	12,348	13,726	15,353	16,217
30	-0,456	13,938	0,084	11,958	12,542	13,938	15,572	16,433
35	-0,383	14,128	0,084	12,122	12,716	14,128	15,767	16,624
40	-0,310	14,290	0,083	12,261	12,864	14,290	15,929	16,781
45	-0,237	14,414	0,083	12,369	12,979	14,414	16,050	16,894
50	-0,164	14,506	0,082	12,448	13,065	14,506	16,136	16,970
55	-0,091	14,585	0,082	12,517	13,139	14,585	16,207	17,032
60	-0,018	14,677	0,081	12,597	13,225	14,677	16,292	17,108
65	0,055	14,786	0,081	12,691	13,326	14,786	16,397	17,205
70	0,128	14,903	0,080	12,792	13,434	14,903	16,510	17,311
75	0,201	15,021	0,080	12,895	13,544	15,021	16,624	17,419
80	0,274	15,141	0,079	12,998	13,655	15,141	16,741	17,529
81	0,289	15,165	0,079	13,019	13,678	15,165	16,764	17,552

S18: LMS parameter modeled percentiles of lean mass/height^2 (kg/m^2) vs. age in females with high fat mass/height^2 (kg/m^2).

**LMI females with high FMI** (n=1435) **age (yrs)**	**L**	**M**	**S**	**3rd**	**10th**	**50th**	**90th**	**97th**
18	-1,376	14,765	0,092	12,638	13,233	14,765	16,798	18,002
20	-1,348	14,880	0,095	12,680	13,294	14,880	16,995	18,253
25	-1,28	15,170	0,101	12,807	13,465	15,170	17,464	18,838
30	-1,211	15,466	0,104	12,978	13,670	15,466	17,883	19,329
35	-1,143	15,767	0,106	13,171	13,894	15,767	18,280	19,778
40	-1,074	16,051	0,106	13,395	14,137	16,051	18,593	20,094
45	-1,006	16,288	0,100	13,713	14,440	16,288	18,680	20,058
50	-0,937	16,468	0,0900	14,083	14,767	16,468	18,594	19,781
55	-0,869	16,581	0,084	14,289	14,952	16,581	18,576	19,670
60	-0,800	16,625	0,085	14,293	14,969	16,625	18,642	19,743
65	-0,732	16,624	0,089	14,196	14,899	16,624	18,727	19,875
70	-0,663	16,590	0,092	14,089	14,814	16,590	18,751	19,928
75	-0,594	16,528	0,091	14,035	14,760	16,528	18,659	19,810
80	-0,526	16,449	0,089	14,021	14,732	16,449	18,492	19,583
81	-0,512	16,433	0,088	14,017	14,725	16,433	18,459	19,539

S19: LMS parameter modeled percentiles of lean mass/height^2 (kg/m^2) for males in different BMI categories.

males	age (yrs)	L	M	S	3rd	10th	50th	90th	97th
**BMI 18.5-<25 kg/m^2** (n=3099)	18	0,245	16,551	0,082	14,147	14,882	16,551	18,357	19,251
	20	0,267	16,568	0,081	14,178	14,91	16,568	18,358	19,241
	30	0,374	16,631	0,078	14,312	15,027	16,631	18,337	19,171
	40	0,48	16,604	0,074	14,368	15,062	16,604	18,224	19,008
	50	0,587	16,505	0,071	14,36	15,03	16,505	18,036	18,772
	60	0,694	16,389	0,068	14,335	14,98	16,389	17,836	18,526
	70	0,801	16,253	0,065	14,289	14,909	16,253	17,619	18,265
	80	0,907	16,133	0,062	14,255	14,851	16,133	17,425	18,032
**BMI ≥25 -<30 kg/m^2** (n=2134)	18	-1,458	18,427	0,078	16,136	16,788	18,427	20,527	21,729
	20	-1,358	18,398	0,077	16,121	16,772	18,398	20,453	21,616
	30	-0,854	18,257	0,072	16,05	16,697	18,257	20,111	21,103
	40	-0,35	18,173	0,068	16,031	16,674	18,173	19,859	20,72
	50	0,154	18,133	0,064	16,053	16,693	18,133	19,677	20,436
	60	0,658	18,026	0,06	16,019	16,649	18,026	19,439	20,112
	70	1,162	17,777	0,057	15,859	16,474	17,777	19,064	19,661
	80	1,666	17,509	0,053	15,683	16,28	17,509	18,683	19,216
**BMI ≥30 kg/m^2** (n=1001)	18	1,162	19,987	0,078	17,009	17,967	19,987	21,975	22,894
	20	0,881	20,009	0,078	17,108	18,026	20,009	22,016	22,962
	30	-0,456	20,133	0,076	17,535	18,307	20,133	22,237	23,332
	40	-1,266	20,196	0,074	17,77	18,468	20,196	22,339	23,531
	50	-1,598	20,076	0,072	17,76	18,42	20,076	22,183	23,384
	60	-1,599	19,827	0,07	17,59	18,23	19,827	21,848	22,994
	70	-1,163	19,354	0,068	17,168	17,805	19,354	21,227	22,245
	80	-0,5	18,735	0,067	16,591	17,232	18,735	20,442	21,322

S20: LMS parameter modeled percentiles of lean mass/height^2 (kg/m^2) for females in different BMI categories.

**females**	**age (yrs)**	**L**	**M**	**S**	**3rd**	**10th**	**50th**	**90th**	**97th**
**BMI 18.5-<25 kg/m^2** (n=3099)	18	-0,376	13,489	0,075	11,759	12,275	13,489	14,873	15,588
	20	-0,317	13,561	0,075	11,822	12,343	13,561	14,944	15,654
	30	-0,024	13,865	0,073	12,085	12,625	13,865	15,229	15,914
	40	0,269	14,051	0,072	12,246	12,801	14,051	15,388	16,044
	50	0,561	14,007	0,07	12,206	12,768	14,007	15,297	15,916
	60	0,854	13,874	0,069	12,088	12,653	13,874	15,11	15,694
	70	1,147	13,825	0,068	12,045	12,616	13,825	15,019	15,572
	80	1,44	13,853	0,067	12,068	12,649	13,853	15,013	15,541
**BMI ≥25 -<30 kg/m^2** (n=1533)	18	-2,745	15,144	0,064	13,652	14,067	15,144	16,612	17,515
	20	-2,622	15,163	0,064	13,663	14,082	15,163	16,622	17,507
	30	-2,004	15,282	0,063	13,736	14,177	15,282	16,694	17,505
	40	-1,386	15,427	0,063	13,83	14,294	15,427	16,8	17,548
	50	-0,768	15,344	0,062	13,716	14,2	15,344	16,662	17,348
	60	-0,15	15,113	0,062	13,468	13,969	15,113	16,367	16,994
	70	0,468	15,049	0,061	13,365	13,89	15,049	16,257	16,839
	80	1,085	15,033	0,061	13,302	13,856	15,033	16,203	16,748
**BMI ≥30 kg/m^2** (n=901)	18	-1,812	17,046	0,068	15,197	15,723	17,046	18,739	19,711
	20	-1,809	17,083	0,07	15,17	15,712	17,083	18,854	19,879
	30	-1,794	17,263	0,083	15,053	15,669	17,263	19,412	20,709
	40	-1,779	17,355	0,084	15,095	15,723	17,355	19,564	20,903
	50	-1,764	17,219	0,075	15,19	15,763	17,219	19,12	20,231
	60	-1,749	16,959	0,073	14,999	15,554	16,959	18,78	19,836
	70	-1,734	16,742	0,077	14,717	15,288	16,742	18,65	19,771
	80	-1,72	16,525	0,075	14,555	15,112	16,525	18,369	19,444

S21: LMS parameter modeled percentiles of appendicular lean mass/height^2 (kg/m^2) for males in different BMI categories.

males	age (yrs)	L	M	S	3rd	10th	50th	90th	97th
**BMI 18.5-<25 kg/m^2** (n=3099)	18	-0,495	7,972	0,103	6,629	7,017	7,972	9,134	9,767
	20	-0,43	7,967	0,102	6,625	7,015	7,967	9,115	9,733
	30	-0,106	7,921	0,099	6,591	6,986	7,921	8,996	9,553
	40	0,219	7,813	0,095	6,506	6,902	7,813	8,815	9,317
	50	0,543	7,661	0,092	6,384	6,779	7,661	8,591	9,042
	60	0,868	7,465	0,089	6,227	6,618	7,465	8,325	8,731
	70	1,193	7,249	0,086	6,053	6,439	7,249	8,043	8,408
	80	1,517	7,047	0,083	5,891	6,271	7,047	7,781	8,112
**BMI ≥25 -<30 kg/m^2** (n=2134)	18	-1,765	8,963	0,102	7,599	7,969	8,963	10,398	11,326
	20	-1,489	8,927	0,101	7,553	7,931	8,927	10,307	11,16
	30	0,069	8,746	0,096	7,289	7,727	8,746	9,888	10,469
	40	0,636	8,582	0,092	7,148	7,595	8,582	9,612	10,109
	50	-0,073	8,428	0,087	7,156	7,537	8,428	9,432	9,944
	60	0,45	8,228	0,083	6,993	7,375	8,228	9,134	9,575
	70	1,676	7,985	0,079	6,723	7,141	7,985	8,773	9,125
	80	1,468	7,752	0,076	6,606	6,981	7,752	8,489	8,823
**BMI ≥30 kg/m^2** (n=1001)	18	0,939	9,723	0,094	8,018	8,559	9,723	10,896	11,447
	20	0,717	9,717	0,094	8,05	8,571	9,717	10,902	11,469
	30	-0,329	9,688	0,093	8,175	8,621	9,688	10,94	11,599
	40	-0,888	9,62	0,092	8,187	8,598	9,62	10,899	11,613
	50	-0,828	9,451	0,091	8,047	8,451	9,451	10,692	11,379
	60	-0,403	9,183	0,091	7,785	8,196	9,183	10,345	10,961
	70	0,175	8,811	0,09	7,418	7,84	8,811	9,878	10,412
	80	0,811	8,385	0,089	6,998	7,434	8,385	9,356	9,816

S22: LMS parameter modeled percentiles of appendicular lean mass/height^2 (kg/m^2) for females in different BMI categories.

**females**	**age (yrs)**	**L**	**M**	**S**	**3rd**	**10th**	**50th**	**90th**	**97th**
**BMI 18.5-<25 kg/m^2** (n=3099)	18	-0,755	6,102	0,106	5,067	5,361	6,102	7,043	7,575
	20	-0,714	6,134	0,106	5,094	5,39	6,134	7,073	7,601
	30	-0,51	6,24	0,104	5,18	5,486	6,24	7,161	7,665
	40	-0,305	6,22	0,102	5,162	5,472	6,22	7,108	7,58
	50	-0,101	6,106	0,1	5,066	5,375	6,106	6,949	7,386
	60	0,103	5,974	0,098	4,955	5,262	5,974	6,772	7,177
	70	0,308	5,91	0,097	4,901	5,208	5,91	6,674	7,054
	80	0,512	5,898	0,095	4,89	5,201	5,898	6,638	6,998
**BMI ≥25 -<30 kg/m^2** (n=2134)	18	-2,164	7,004	0,087	6,09	6,34	7,004	7,954	8,565
	20	-2,097	6,999	0,087	6,083	6,333	6,999	7,943	8,545
	30	-1,763	6,975	0,087	6,044	6,303	6,975	7,892	8,452
	40	-1,429	6,946	0,087	6,001	6,267	6,946	7,837	8,359
	50	-1,095	6,849	0,086	5,898	6,169	6,849	7,707	8,191
	60	-0,762	6,696	0,086	5,746	6,022	6,696	7,516	7,963
	70	-0,428	6,577	0,086	5,623	5,904	6,577	7,365	7,78
	80	-0,094	6,486	0,086	5,523	5,812	6,486	7,246	7,635
**BMI ≥30 kg/m^2** (n=1001)	18	2,086	8,022	0,098	6,352	6,932	8,022	8,971	9,379
	20	1,433	8,029	0,098	6,477	6,987	8,029	9,015	9,46
	30	-1,014	8,059	0,099	6,8	7,156	8,059	9,224	9,894
	40	-1,182	8,025	0,099	6,785	7,133	8,025	9,204	9,896
	50	-0,789	7,858	0,099	6,606	6,964	7,858	8,981	9,609
	60	-0,241	7,636	0,099	6,364	6,739	7,636	8,688	9,241
	70	-1,648	7,479	0,099	6,356	6,663	7,479	8,628	9,35
	80	-0,564	7,34	0,099	6,144	6,49	7,34	8,378	8,945
